# Association study of *ESR1* rs9340799, rs2234693, and *MMP2* rs243865 variants in Iranian women with premature ovarian insufficiency: A case-control study

**DOI:** 10.18502/ijrm.v20i10.12268

**Published:** 2022-11-02

**Authors:** Farzaneh Sadat Eshaghi, Masoud Dehghan Tezerjani, Nasrin Ghasemi, Mohammadreza Dehghani

**Affiliations:** ^1^Department of Genetics, Faculty of Medicine, Shahid Sadoughi University of Medical Sciences, Yazd, Iran.; ^2^Abortion Research Center, Yazd Reproductive Sciences Institute, Shahid Sadoughi University of Medical Sciences, Yazd, Iran.; ^3^Medical Genetics Research Center, Shahid Sadoughi University of Medical Sciences, Yazd, Iran.

**Keywords:** Matrix metalloproteinase-2, Estrogen receptor alpha, Primary ovarian insufficiency, Female infertility.

## Abstract

**Background:**

Primary ovarian insufficiency (POI) is a rare disease clinically characterized by ovarian follicles depletion or dysfunction and menopause before the age of 40 yr as the cut-off age for POI. It is a complex disease, and its etiology involves several factors. However, genetic factors have a predominant role in the susceptibility to the disease.

**Objective:**

This study aims to investigate the polymorphisms of rs243865 in the matrix metallopeptidase 2 (*MMP2*) gene and rs2234693 and rs9340799 in the estrogen receptor 1 *(ESR1*) gene with susceptibility to POI in Iranian women under 35 yr.

**Materials and Methods:**

This case-control study was performed on 150 women with POI and 150 healthy women who were referred to Yazd Reproductive Sciences Institute, Yazd, Iran between May-October 2020. The genotyping of *ESR1* rs9340799, rs2234693, and *MMP2* rs243865 polymorphism was done using tetra-amplification refractory mutation system-polymerase chain reaction. In addition, haplotype analysis and linkage disequilibrium were investigated by SNPanalyzer software.

**Results:**

Our study revealed the frequency of rs243865 TT, CC genotypes in the *MMP2* gene and rs2234693 CC, TT; and rs9340799 GG, AA in the *ESR1* gene were more prevalent in the case group compared to the control group. In addition, *ESR1* rs2234693 and rs9340799 genotypes showed significant association with the development of the disease in our population. Among 4 haplotypes for 2 polymorphisms in the *ESR1* gene, rs2234693T/rs9340799A haplotype was associated with conferring risk to POI.

**Conclusion:**

*ESR1* rs2234693 and rs9340799 polymorphism were strongly associated with our population's POI.

## 1. Introduction

Primary ovarian insufficiency (POI) is a rare disease with hyper gonadotrophic amenorrhea in women before the age of 40 yr (1). It occurs in around 1/1000 women < 30 yr and 1/10000 < 20 yr (2). The main causes of POI in most women are still unknown; however, genetic reasons (chromosomal abnormalities and gene mutations), infections, and metabolic and autoimmunity disorders are associated with developing POI (3, 4). This condition is clinically characterized by amenorrhea, decreased levels of estradiol (E2) and anti-Mullerian hormone (AMH); and an increased level of luteinizing hormone (LH) and follicle-stimulating hormone (FSH).

Several studies suggested a possible association between matrix metallopeptidase 2 (*MMP2*) genetic variants and vulnerability to POI. This gene on chromosome 16q13-21 has 17 exons and belongs to the *MMP2* gene family involved in breaking down signal transduction molecules and extracellular matrix components (5-7). The expression of *MMP2* has been identified in both the testis and ovary. *MMP2* is localized to the oogonium/oocyte cytoplasm in the ovary, with varying intensities. In addition, it was detected in the ovarian stroma (8).

Different studies have also investigated the association of polymorphism in the estrogen receptor 1 (*ESR1*) gene with developing POI (9); however, their results are controversial in different populations. This gene located on 6q25.1-q25.2 with 23 exons, encodes a ligand-activated transcription factor as well as an estrogen receptor. The receptor has a fundamental role in the pathogenesis of endometrial cancer, breast cancer, and osteoporosis. rs2234693 and rs9340799 polymorphism in *ESR1* is the most studied variant in women suffering from POI. Although rs2234693 polymorphism is located in the intronic section of the *ESR1* gene, it was reported to be associated with normal menopause in Korean and Dutch women (10). This polymorphism was also investigated in Chinese, Brazilian, and European women, and the results revealed a significant association with the onset of POI (11-13). The other polymorphism in this gene, rs9340799, decreases the risk of developing POI in the Korean population. However, no association of this polymorphism was found with Chinese and Brazilian women (11, 12, 14).

This study aimed to evaluate the association of rs243865 polymorphism in the *MMP2* gene and rs2234693 and rs9340799 polymorphisms in the *ESR1* gene with the risk of POI in women under 35 yr.

## 2. Materials and Methods

### Sample collection

Hundred and fifty women women with POI and 150 healthy women as a control group were involved in this case-control study. The samples were collected from Yazd Reproductive Sciences Institute, Yazd, Iran from May-October 2020. POI women were selected based on FSH measurements of > 40 mIU/ml; and AMH < 2 ng/ml. Inclusion criteria for healthy participants were negative autoantibodies (anti-ovarian thyroid, antinuclear antibodies), regular menstrual cycles, and at least one live birth. Women with pelvic surgery, positive for autoantibodies, a history of cancer, radiation exposure, and genetic syndrome were excluded.

### Hormonal evaluation

LH, E2, AMH, thyroid stimulating hormone, prolactin, and FSH have been evaluated in both groups using the Pishtaz Teb kit (Pishtaz Teb, Iran) on the Stat Fax system (Awareness Technology, USA). Samples were collected on the 3 ${}^{\text{rd}}$ or 4 ${}^{\text{th}}$ days of menstrual cycles in the control group.

### DNA extraction and polymerase chain reaction (PCR)

DNA was extracted from blood samples of individuals using a DNA extraction kit (Simbiolab, Iran) according to the manufacturer's instructions. The quality of the extracted genomic DNA was evaluated using agarose gel electrophoresis, and the quantity of the samples were checked by a Nanodrop (Thermo Scientific, Wilmington, DE). Then, we used Tetra-primer amplification refractory mutation system PCR to genotype the 3 polymorphisms (15, 16) (Table I).

Each vial of PCR for the rs2234693 polymorphism in the *ESR1* gene contained 1 ml of DNA, (0.4 μL of FO+0.4 μL RO+1 ml FI+1 μL RI) primers, 12 ml of master mix (Amplicon), and 9.2 μL of water in a final volume of 25 μL. The vial for testing the rs9340799 polymorphism in the *ESR1* included 1 μl of DNA, (0.5 μL of FO+0.5 μL RO+1.2 μL FI + 1.2 μL RI) primers, 12 μL of master mix (Amplicon), and 8.6 μL of water in a final volume of 25 μL. The volume of each vial for the rs243865 polymorphism in the *MMP2* gene is 10 μL master mix, 0.4 μL external forward primer, 0.4 μL external reverse primer, 0.8 μL internal forward primer, 0.8 μL internal reverse primer, 6.6 ml of water, and 1 μL of DNA in a final volume of 20 μL.

The PCR condition was done by following steps: 95 C for 10 min as initial denaturation, denaturation at 95 C for 30 sec, annealing at 64.5 C (rs243865), 62 C (rs2234693), 61 C (rs9340799) for 30 sec, and extension at 72 C for 30 sec (for 38 cycles) and final extension at 72 C for 5 min. Next, the PCR products were loaded on 2% agarose gel. To check the genotyping quality, we sequenced all polymorphisms in random samples bidirectionally.

**Table 1:** Primers used for genotyping of polymorphisms and their amplicons


	**Sequence (5 ' → 3 ' )**	**Length of products**
***ESR1***			
	**F outer**	CAGGGTTATGTGGCAATGACG	368bp (internal control)
	**R outer**	ATTACCTCTTGCCGTCTGTTGC	
	**F inner 223**	ATCTGAGTTCCAAATGTCCCATCT	293bp (wild type, T allele)
	**R inner 223**	GGGAAACAGAGACAAAGCATAAACCG	124bp (mutant, C allele)
	**F inner 934**	CCAGAGACCCTGAGTGTGGTATG	246bp (mutant, A allele)
	**R inner 934**	ACCAATGCTCATCCCAACGCT	165bp (wild type, G allele)
***MMP2***			
	**F outer**	TTCTCAAACTGTTCCCTGCTGACCC	
	**R outer**	ACGCCTGACTTCAGCCCCTAAACTAG	305bp (internal control)
	**F inner**	CCATATTCCCCACCCAGCACGCT		119bp (mutant, T allele)
	**R inner**	GAGCTGAGACCTGAAGAGCTAAAGAGTTG	238bp (wild type, C allele)
F: Forward, R: Reverse, bp: Base pair, *ESR1*: Estrogen receptor 1, *MMP2*: Matrix metallopeptidase 2			

### Ethical considerations

The study was approved by the local ethics committee of the Shahid Sadoughi University of Medical Sciences, Yazd, Iran (Code: IR.SSU.MEDICINE.REC.1399.030). Written informed consent was also obtained from all the participants.

### Statistical analysis

The Statistical Package for the Social Sciences, version 21, SPSS Inc, Chicago, Illinois, USA software was applied to analyze the data. For the evaluation of clinical features for healthy and POI individuals, we calculated the p-values by independent 2-sample *t* test. The difference in genotypes and allele frequency between the control and case groups were also investigated by Fisher's exact test. To analyze the strength of the association between the genotypes/alleles of the polymorphism and susceptibility to POI, the odds ratio (OR) and their 95% confidence intervals (95% CI) were estimated. Age was considered a covariate, and its effects were removed from the analysis. We considered p-value < 0.05 as a significant value. In addition, an SNPanalyzer (v2) was employed for haplotype and linkage disequilibrium analyses.

## 3. Results

The clinical features and characteristics of the participants are described in table II. The FSH, LH, and E2 showed a significant difference between the case and control groups (p < 0.001). However, no significant differences were found for other clinical features.

**Figure 1:**
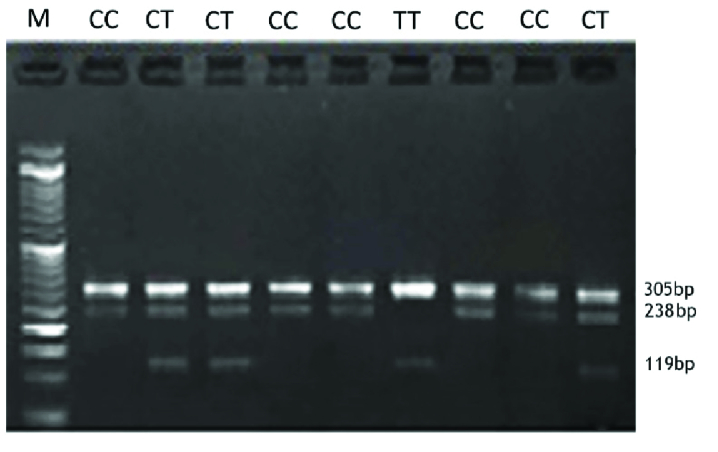
Visualization of ARMS-PCR products on agarose gel for rs243865 in the *MMP2* gene. M: DNA marker. Product sizes were 238bp for the C allele, 119bp for the T allele, and 305bp for 2 outer primers (control band).

**Figure 2:**
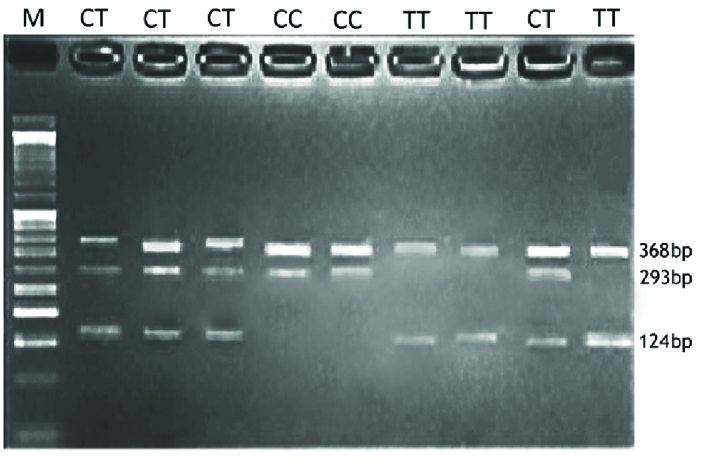
Visualization of ARMS-PCR products on agarose gel for rs2234693 in *ESR1* gene. M: DNA marker. Product sizes were 124bp for the C allele, 293bp for the T allele, and 368bp for the control band.

**Figure 3:**
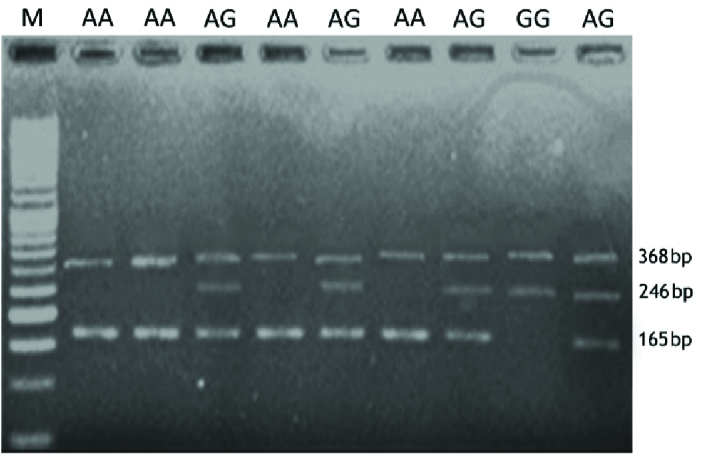
Visualization of ARMS-PCR products on agarose gel for rs9340799 in *ESR1* gene. M: DNA marker. Product sizes were 246bp for the A allele, 165bp for the G allele, and 368bp for the control band.

The visualization of amplification refractory mutation system PCR products on agarose gel for* MMP2* -rs243865, *ESR1*-rs2234693, and *ESR1*-rs9340799 are shown in figures 1, 2, and 3, respectively.

The genotype frequencies and ORs for *MMP2* -rs243865, *ESR1*-rs2234693, and *ESR1*-rs9340799 are also shown in tables III, IV, and V, respectively. Our study shows the frequency of rs243865 TT, CC genotypes in the *MMP2* gene and rs2234693 CC, TT; and rs9340799 GG, AA in the *ESR1* gene were more prevalent in the case group compared to the control group. There was a significant association of *ESR1* rs2234693 and rs9340799 genotypes with the disease in our population (p < 0.001). No significant association was found for these 3 polymorphisms at the allelic level. Analysis of different models of inheritance revealed that the codominant model (CT vs. CC+TT) and CT vs. CC for rs243865 polymorphism (Table III); and dominant model (TC+CC vs. TT), recessive model (CC vs. TT+TC), codominant model (TC vs. TT+CC) and TC vs. TT (WM vs. WW) for rs2234693 polymorphism (Table IV); and dominant model (AG+GG vs. AA), recessive model (GG vs. AA+AG), codominant model (AG vs. AA+GG) and AG vs. AA (WM vs. WW) for rs9340799 polymorphism (Table V) were significantly different between the case and control group.

The combinational analysis also revealed that rs243865 CC/rs2234693 CC/rs9340799 AA, rs243865 CC/rs2234693TC/rs9340799 AG, rs243865 CC/rs2234693 TT/rs9340799 AA, rs243865 CC/rs2234693 TT/rs9340799 GG, and rs243865 CT/rs2234693 TC/rs9340799 AG genotypes were significantly associated with the susceptibility to POI in our population (table VI). Among the 4 haplotypes for 2 polymorphisms in the *ESR1* gene, rs2234693T/rs9340799A haplotype was found to be associated with conferring risk to POI (p = 0.04, OR = 1.42) (Table VII). Pairwise LD analysis also showed a moderate LD (D ' :30) for *ESR1* -351 A/G (rs9340799) and *ESR1* -397 T/C (rs2234693) polymorphisms in our population.

**Table 2:** Clinical features of participants


**Features**	**Control**	<statement> <title>Case </title> </statement>	**Total**	P-value*
**Age **	28.87 ± 4.45	30.03 ± 4.05	29.45 ± 4.29	
**AMH**	3.4 ± 0.9	0.82 ± 0.46	2.11 ± 1.47	0.97
**TSH**	4.18 ± 3.83	3.75 ± 3.59	3.96 ± 3.72	0.31
**Anti. Tpo**	33.1 ± 64.85 (29 ${}^{\text{a}}$ )	60.06 ± 144.47 (30.2 ${}^{\text{a}}$ )	46.58 ± 112.6 (29.53 ${}^{\text{a}}$ )	0.05
**Prolactin**	20.26 ± 47.53 (15 ${}^{\text{a}}$ )	25.57 ± 48.32 (13.4 ${}^{\text{a}}$ )	22.92 ± 47.92 (14.9 ${}^{\text{a}}$ )	0.34
**FSH**	7.28 ± 6.69	11.51 ± 10.48	9.4 ± 9.03	< 0.001
**LH**	6.26 ± 4.54	17.61 ± 10.73	11.93 ± 9.99	< 0.001
**E2**	76.34 ± 66.08	12.07 ± 9.87	44.2 ± 57.1 (44.32 ${}^{\text{a}}$ )	< 0.001
Data presented as Mean ± SD. *P-values were calculated by independent 2-sample *t* test, POI: Primary ovarian insufficiency, AMH: Anti-Mullerian hormone, TSH: Thyroid stimulating hormone, Anti. Tpo: Anti thyroid peroxidase, FSH: Follicle-stimulating hormone, LH: Luteinizing hormone, E2: Estradiol, ${}^{\text{a}}$ Interquartile range (IQR)				

**Table 3:** Association analysis of *MMP2* rs243865 polymorphism with risk of endometriosis, according to multiple inheritance models


**Model**	**Types **	**Control**	<statement> <title>Case </title> </statement>	**Total**	P-value*	**OR (95% CI)**
	CC	115 (76.67)	128 (85.33)	243 (81)	ref	ref
**Dominant**	CT+TT	35 (23.33)	22 (14.67)	57 (19)	0.07	0.57 (0.31-1.03)
		CC+CT	149 (99.33)	148 (98.67)	297 (99)	ref	ref
**Recessive**	TT	1 (0.67)	2 (1.33)	3 (1)	0.55	2.08 (0.19-45.24)
		CC+TT	116 (77.33)	130 (86.67)	246 (82)	ref	ref
**Codominant**	CT	34 (22.67)	20 (13.33)	54 (18)	0.04	0.53 (0.28-0.97)
		CC	115 (76.67)	128 (85.33)	243 (81)	ref	ref
**WM vs. WW**	CT	34 (22.67)	20 (13.33)	54 (18)	0.04	0.53 (0.28-0.98)
		CC	115 (76.67)	128 (85.33)	243 (81)	ref	ref
**MM vs. WW**	TT	1 (0.67)	2 (1.33)	3 (1)	0.62	1.84 (0.17-40.04)
		C	264 (88)	276 (92)	540 (90)	ref	ref
**Allelic**	T	36 (12)	24 (8)	60 (10)	0.12	0.64 (0.37-1.11)
	Result of genotypes analysis of *MMP2* rs243865 polymorphism in different models of inheritance, data presented as n (%). *adjusted p-value for different models and calculated based on Fisher's exact test, M: Mutant, W: Wild type,* MMP2*: Matrix metallopeptidase 2, ref: Considered as a reference						

**Table 4:** Association analysis of *ESR1* rs2234693 polymorphism with risk of endometriosis, according to multiple inheritance models


**Model**	**Types **	**Control**	<statement> <title>Case </title> </statement>	**Total**	P-value*	**OR (95% CI)**
	TT	40 (26.67)	68 (45.33)	108 (36)	ref	ref
**Dominant**	TC+CC	110 (73.33)	82 (54.67)	192 (64)	< 0.001	0.45 (0.27-0.73)
		TT+TC	138 (92)	114 (76)	252 (84)	ref	ref
**Recessive**	CC	12 (8)	36 (24)	48 (16)	< 0.001	3.55 (1.80-7.44)
		TT+CC	52 (34.67)	104 (69.33)	156 (52)	ref	ref
**Codominant**	TC	98 (65.33)	46 (30.67)	144 (48)	< 0.001	0.24 (0.14-0.39)
		TT	40 (26.67)	68 (45.33)	108 (36)	ref	ref
**WM vs. WW**	TC	98 (65.33)	46 (30.67)	144 (48)	< 0.001	0.28 (0.16-0.47)
		TT	40 (26.67)	68 (45.33)	108 (36)	ref	ref
**MM vs. WW**	CC	12 (8)	36 (24)	48 (16)	0.14	1.77 (0.83-3.95)
		T	178 (59.33)	182 (60.67)	360 (60)	ref	ref
**Allelic**	C	122 (40.67)	118 (39.33)	240 (40)	0.77	0.95 (0.68-1.32)
	Result of genotypes analysis of *ESR1* rs2234693 polymorphism in different models of inheritance. Data presented as n (%). *adjusted p-value for different models and calculated based on Fisher's exact test, M: Mutant, W: Wild type,* ESR1*: Estrogen receptor 1, ref: Considered as a reference						

**Table 5:** Association analysis of *ESR1* rs9340799 polymorphism with risk of endometriosis, according to multiple inheritance models


**Model**	**Types**	**Control**	<statement> <title>Case </title> </statement>	**Total**	P-value*	**OR (95% CI)**
	AA	60 (40)	86 (57.33)	146 (48.67)	ref	ref
**Dominant**	AG+GG	90 (60)	64 (42.67)	154 (51.33)	< 0.001	0.48 (0.30-0.77)
		AA+AG	144 (96)	128 (85.33)	272 (90.67)	ref	ref
**Recessive**	GG	6 (4)	22 (14.67)	28 (9.33)	< 0.001	4.19 (1.73-11.73)
		AA+GG	66 (44)	108 (72)	174 (58)	ref	ref
**Codominant**	AG	84 (56)	42 (28)	126 (42)	< 0.001	0.29 (0.18-0.48)
		AA	60 (40)	86 (57.33)	146 (48.67)	ref	ref
**WM vs. WW**	AG	84 (56)	42 (28)	126 (42)	< 0.001	0.34 (0.20-0.56)
		AA	60 (40)	86 (57.33)	146 (48.67)	ref	ref
**MM vs. WW**	GG	6 (4)	22 (14.67)	28 (9.33)	0.05	2.62 (1.03-7.58)
		A	204 (68)	214 (71.33)	418 (69.67)	ref	ref
**Allelic**	G	96 (32)	86 (28.67)	182 (30.33)	0.36	0.84 (0.59-1.20)
	Result of genotypes analysis of *ESR1* rs9340799 polymorphism in different models of inheritance. Data presented as n (%). *adjusted p-value for different models and calculated based on Fisher's exact test, M: Mutant, W: Wild type,* ESR1*: Estrogen receptor 1, ref: Considered as a reference						

**Table 6:** The combinatorial analysis of *MMP2* and *ESR1* polymorphisms in case and control groups


**Genotype**	**Control**	<statement> <title>Case </title> </statement>	**Total**	**P-value ${}^{\text{a}}$ **	**OR (95% CI)**
**CC/CC/AA**	7 (4.67)	24 (16)	31 (10.33)	< 0.001	3.89 (1.62-9.33)
**CC/CC/AG**	4 (2.67)	5 (3.33)	9 (3)	1.00	1.25 (0.33-4.78)
**CC/CC/GG**	0 (0)	3 (2)	3 (1)	0.25	-
**CC/TC/AA**	27 (18)	23 (15.33)	50 (16.67)	0.64	0.82 (0.44-1.51)
**CC/TC/AG**	42 (28)	11 (7.33)	53 (17.67)	< 0.001	0.20 (0.10-0.41)
**CC/TC/GG**	3 (2)	3 (2)	6 (2)	1.00	1.00 (0.19-5.03)
**CC/TT/AA**	12 (8)	26 (17.33)	38 (12.67)	0.02	2.41 (1.16-4.98)
**CC/TT/AG**	18 (12)	20 (13.33)	38 (12.67)	0.86	1.12 (0.57-2.22)
**CC/TT/GG**	2 (1.33)	13 (8.67)	15 (5)	< 0.001	7.02 (1.55-31.68)
**CT/CC/AA**	0 (0)	2 (1.33)	2 (0.67)	0.45	-
**CT/CC/AG**	1 (0.67)	0 (0)	1 (0.33)	1.00	0.00 (0.00-NaN)
**CT/CC/GG**	0 (0)	2 (1.33)	2 (0.67)	0.45	-
**CT/TC/AA**	11 (7.33)	5 (3.33)	16 (5.33)	0.20	0.43 (0.14-1.28)
**CT/TC/AG**	13 (8.67)	2 (1.33)	15 (5)	< 0.001	0.14 (0.03-0.64)
**CT/TC/GG**	1 (0.67)	0 (0)	1 (0.33)	1.00	0.00 (0.00-NaN)
**CT/TT/AA**	3 (2)	5 (3.33)	8 (2.67)	0.72	1.68 (0.39-7.20)
**CT/TT/AG**	5 (3.33)	4 (2.67)	9 (3)	1.00	0.79 (0.20-3.01)
**TT/TC/AA**	0 (0)	1 (0.67)	1 (0.33)	1.00	-
**TT/TC/AG**	1 (0.67)	0 (0)	1 (0.33)	1.00	0.00 (0.00-NaN)
**TT/TC/GG**	0 (0)	1 (0.67)	1 (0.33)	1.00	-
Result of 20 combinatorial analysis of 9 genotypes from 3 SNPs in *MMP2* and *ESR1* genes, and each row shows the combination of 3 different genotypes from 3 SNPs. Data presented as n (%). Odds ratio and p-value have been calculated for combined genotypes of different genotypes. ${}^{\text{a}}$ Calculated based on Fisher's exact test, *MMP2*: Matrix metallopeptidase 2,* ESR1*: Estrogen receptor 1, NaN: Not a number					

**Table 7:** Haplotypes analysis of *ESR1* gene polymorphism


**Haplotype**	**x^2^**	**P-value ${}^{\text{a}}$ **	**OR (95% CI)**
**T/A**	4.258	0.04	1.42 (1.01-2.00)
**C/A**	1.43	0.23	0.81 (0.58-1.14)
**T/G**	3.494	0.06	0.70 (0.48-1.01)
**C/G**	3.746	0.05	2.18 (0.97-4.91)
Results of analysis for 4 different haplotypes of 2 SNPs in the *ESR1* gene calculated by SNP analyzer (v2) software. ${}^{\text{a}}$ Calculated by Chi-square test, * ESR1*: Estrogen receptor 1			

## 4. Discussion

Like other multifactorial diseases, POI originated from various genetics and environmental factors (1). Therefore, we performed a case-control study to investigate the possible association of 3 polymorphisms in the *MMP2* and *ESR1* gene and susceptibility to POI in Iranian women. Our study showed a significant association of ESR1 rs2234693 and rs9340799 genotypes with the disease in our population. In addition, the codominant model (CT vs. CC+TT) and CT vs. CC of *MMP2* rs243865 polymorphism were found to be correlated with the risk of POI. No allelic association was found for these 3 polymorphisms. In the *ESR1* gene, rs2234693T/rs9340799A haplotype was also associated with developing vulnerability to POI.

The *ESR1* gene encoding estrogen α receptor is one of the fundamental molecules in the human reproductive system (17). Estrogen has a positive or negative impact on the regulation of gonadotropin secretion, folliculogenesis, and ovulation. Therefore, any variations in its gene may affect the time of menarche or menopause, resulting in developing POI (17, 18). In addition, the polymorphisms in this gene have been investigated in different diseases related to females, such as breast cancer, endometriosis, and uterine fibroid (19-21). Although some studies revealed the positive association of *ESR1* -397 T/C (rs2234693) and -351 A/G (rs9340799) with POI (22, 23), others showed conflicting results (24, 25). Similar to our study, a study on Caucasian women revealed a positive association of these 2 polymorphisms with POI (23). In addition, a significant association of these 2 polymorphisms and their haplotype with POI was found in the Chinese population (22). However, a meta-analysis study showed no association of these polymorphisms with susceptibility to POI (26). In contrast to our study, a study on Korean women also found no association of *ESR1* -397 T/C (rs2234693) with the disease (27). Following our study, another meta-analysis investigating these 2 polymorphisms (3 case-control studies with 1396 subjects) revealed a significant association of *ESR1* -397 T/C (rs2234693) polymorphism with POI in the Asian population in all models; however, no significant association was found for any models in the European population. In terms of -351 A/G (rs9340799) polymorphism, this meta-analysis showed no association overall, but under dominant model was associated with POI in the Asian population (18). A functional study also indicated that CC genotypes of rs2234693 significantly reduces the *ESR1* expression level (28). C allele decreases the binding of AP-4 as a transcriptional activator to the sense strand and the binding of ZNF238 as a transcriptional repressor to the antisense strand, resulting in unstable *ESR1* mRNA (29, 30).

*MMP2* is involved in the AMH regression pathway and indirectly reduces AMH in POI women. Genetic variants in this gene, including 1306C > T, may affect the expression of this gene, and any changes in its expression can lead to ovarian structural and follicular growth changes (7). A functional study showed that the T allele in the SP1 sequence, a consensus sequence in the *MMP2* promoter (-1306 site; ${}^{-1307C}$ (C/T) ACC ${}^{-1303}$ ) can disrupt the activity of the promoter and lower *MMP2* gene expression (31). Therefore, evaluating the polymorphism can pave the way for finding the disease pathogenesis and genes involved in its development. There is only one study investigating the association of *MMP2* -1306C > T rs243865 with POI. Contrary to our study in which only the CT vs. CC model is related to POI, Kim and colleagues indicated that *MMP2* -1306CT + TT was associated with POI susceptibility (7).

To the best of our knowledge, this is the first study investigating the association of these 3 polymorphisms with POI in the Iranian population. Although, more functional studies with more samples and more polymorphisms in these genes can provide more strong results.

## 5. Conclusion

This study indicated that rs2234693 and rs9340799 polymorphisms of the *ESR1* gene and *MMP2* -1306C > T rs243865 in the codominant model are significant in relation to vulnerability to POI among Iranian women. Finding genotypes in susceptibility to POI would help detect POI and provide precious information for counseling of pregnancy loss in the young couple.

##  Conflict of Interest

The authors declare that they do not have any conflict of interest.
